# Fibronectin-targeted dual-acting micelles for combination therapy of metastatic breast cancer

**DOI:** 10.1038/s41392-019-0104-3

**Published:** 2020-02-07

**Authors:** Zhuoran Gong, Min Chen, Qiushi Ren, Xiuli Yue, Zhifei Dai

**Affiliations:** 1 Department of Biomedical Engineering, College of Engineering, Peking University, Beijing, 100871 China; 2 School of Environment, Harbin Institute of Technology, Harbin, 150090 China

**Keywords:** Metastasis, Breast cancer

## Abstract

Stage IV breast cancer, which has a high risk of invasion, often develops into metastases in distant organs, especially in the lung, and this could threaten the lives of women. Thus, the development of more advanced therapeutics that can efficiently target metastatic foci is crucial. In this study, we built an dual-acting therapeutic strategy using micelles with high stability functionalized with fibronectin-targeting CREKA peptides encapsulating two slightly soluble chemotherapy agents in water, doxorubicin (D) and vinorelbine (V), which we termed C-DVM. We found that small C-DVM micelles could efficiently codeliver drugs into 4T1 cells and disrupt microtubule structures. C-DVM also exhibited a powerful ability to eradicate and inhibit invasion of 4T1 cells. Moreover, an in vivo pharmacokinetics study showed that C-DVM increased the drug circulation half-life and led to increased enrichment of drugs in lung metastatic foci after 24 h. Moreover, dual-acting C-DVM treatment led to 90% inhibition of metastatic foci development and reduced invasion of metastases. C-DVM could potentially be used as a targeted treatment for metastasis and represents a new approach with higher therapeutic efficacy than conventional chemotherapy for stage IV breast cancer that could be used in the future.

## Introduction

Nowadays, breast cancer has become one of the highly risky cancers threatening women's lives with high incidence. About 6% of breast cancer patients are diagnosed at stage IV, and the survival rate is less than 30%.^1^ Stage IV breast cancer is highly invasive with frequent metastasis to distant sites.^2,3^ Lung metastases are particularly dangerous, and patients showing metastasis to the lungs have nearly seventy percent death rate generally.^4^ Therefore, it is crucial to develop an effective remedy to inhibit the metastases of breast cancer.

Traditional chemotherapeutics is the standard clinic treatment of stage IV breast cancer, whereas the chemotherapeutic agents have deficiencies in long-term prognosis.^5^ As the first choice for the treatment of cancers,^6^ chemotherapy agents generally suffer low delivery efficiency to the tumor site with significant variation among different patients.^7^ That is because of the resistance of metastatic site leading to low-efficiency therapeutic effect exists in cytotoxic agents, which cannot be delivered to metastatic sites precisely.^8,9^ Therefore, advances in breast cancer treatment require new platforms that can shrink the primary tumor, and target metastases by targeted drug delivery.

Cancer cells have the unchecked ability to divide. Microtubules are key components of the cytoskeleton and play a crucial role in mitotic cell division.^10,11^ Antimitotic vinca alkaloids, such as vinblastine,^12–14^ vinorelbine,^15–18^ and vincristine,^19–21^ were developed to inhibit cancer cell growth by targeting microtubules.^22^ These diverse classes of microtubule-targeting agents have long circulation retention, making them a powerful mitosis inhibitor for antitumor treatments.^11^ Besides, clinical combinations of more than one antimitotic drug^23–25^ can improve the efficacy with the reduction of side effects.^10^ It means that improving cancer therapy efficiency concentrating on the target of microtubules polymerization is significant.

Another key component is doxorubicin, which could inhibit the biosynthesis of DNA, a routinely used common chemotherapy drug.^26^ Numerous clinical studies have combined vinorelbine with doxorubicin^27–30^ for breast cancer therapy. However, the survival rate for free vinorelbine and doxorubicin or doxorubicin alone in metastatic breast cancer were low^31^ owing to low penetration and limited distribution of agents in the tumor site.^31,32^ Therefore, improving chemotherapeutic agent enrichment in metastatic foci is crucial.

The tumor microenvironment also has a noteworthy effect on antitumor drug activity.^32,33^ The tumor stroma, containing many extracellular matrix (ECM) proteins, is essential for tumor growth and progression.^34^ Among ECM proteins, fibronectin, a class of adhesive glycoproteins, plays a major role in ECM functions of cancer cells such as cell adhesion, proliferation, and migration.^35^ Moreover, the invasive or metastatic sites consist of the high expression of fibronectin and its complexes, relatively higher than primary tumor sites.^36,37^ Fibronectin has been investigated as a target protein for diagnosis high-risk micro-metastasis of breast cancer.^38^ Targeted delivery of therapeutic drugs to highly fibronectin-expressing metastatic tumor sites may be an effective way to inhibit metastatic invasion.

PE-PEG, a block copolymer, has been widely used in liposome formulations.^39–41^ It has been reported that PE-PEG micelles are an ideal carrier of anti-cancer drugs because of their stability and ability to prolong the circulation time in the bloodstream while increasing the solubility of poorly soluble drugs effectively.^42^ Due to enhanced permeability and retention (EPR) effects, self-assembled drug-loaded micelles formed by amphipathic components can be passively accumulated in tumors but often suffer from low efficiency.^43,44^ Combining micelles with active targeting may be a way of improving chemotherapeutic enrichment efficiency.

Cys-Arg-Glu-Lys-Ala (CREKA), a small pentapeptide, was identified to specifically bind fibrin and the tumor stroma,^45–47^ which meant that CREKA could be used for tumor diagnosis^48,49^ and could play a crucial role in the passive targeting of drugs in the delivery process.^45,50^

**Scheme 1:**
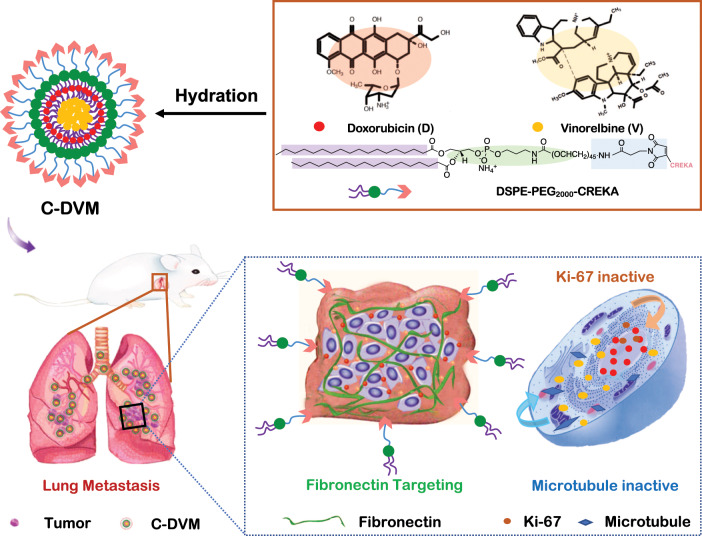
Schematic illustration of the dual-acting combination therapy based on C-DVM for the suppression of development and invasion in breast cancer lung metastatic foci

To build an advanced drug delivery system aimed at overcoming the limitations of traditional chemotherapeutics, in this study, we rationally fabricated a polymeric PEGylated micelle encapsulating doxorubicin and vinorelbine that was functionalized with CREKA, which we termed C-DVM (Scheme 1). In this case, each component performed its function. The CREKA peptides served to target the fibronectin-expressing tumor area consist of high stability and superior dispersibility micelles. The doxorubicin assigned to restricting cell proliferation and the vinorelbine could bind to the tubulin, preventing the microtubule formation during mitosis simultaneously, which we named ‘two-pronged treatment with one targeting’ strategies. Thus, synergetic chemotherapeutic treatment was achieved by drug codelivery for the inhibition metastasis in breast cancer metastatic model. C-DVM achieved the efficient delivery of two chemotherapeutic drugs to the metastatic site due to its small size and targeting. Meanwhile, C-DVM showed outstanding potential for inhibitory invasion of breast cancer both in vitro and in vivo owing to its ‘two-pronged treatment with one targeting’ strategies.

## Results

### Characterization of C-DVM micelle

**Fig. 1:**
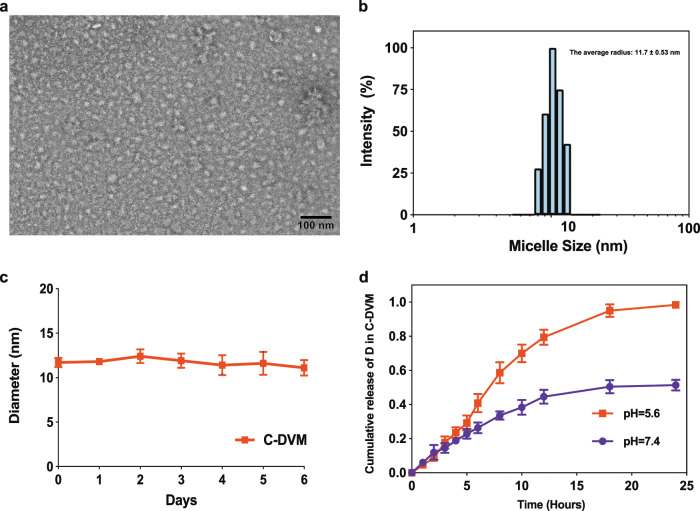
Characterization of C-DVM. **a** The TEM image and (**b**) DLS size distribution demonstrate the uniform synthesis of C-DVM. **c** Stability test of C-DVM over six days in PBS shows little fluctuation in micelle size. **d** Drug release behavior of C-DVM shows pH-sensitive release in acidic environments

We incorporated doxorubicin (D) and vinorelbine (V) into micelles composed of DSPE-PEG-2000 (DVM) and DSPE-PEG-2000-CREKA (C-DVM) through a series of self-assembly steps. The micelles had a high entrapment efficiency (EE) of 98.5% for D and 96.8% for V at a molar ratio of D to V to DSPE-PEG-2000-CREKA of 1:1:2. Transmission electron microscopy (TEM) imaging showed that the spherical C-DVM particles were monodispersed, with a diameter of 15 nm on average (Fig. 1a). Atomic force microscopy (AFM) imaging showed that the micelles were uniform in size, in accordance with the TEM results (Fig. S1). Furthermore, dynamic light-scattering (DLS) measurements (Table 1, Fig. 1b) indicated that DVM and C-DVM exhibited a small and uniform size with a mean diameter of approximately 10 nm and a low polydispersity index (PDI) below 0.3. Because the loaded drugs contained positively charged groups, which was in accordance with expectations, the zeta potential of C-DVM was increased to −7.68 mV compared with −19.37 mV for CREKA-PEG-micelles without loaded drug (C-PM) (Table 1). A similar result was shown between DVM and DSPE-PEG-micelle without loading drug (PM) (Table 1). The critical micelle concentration (CMC) of DSPE-PEG-2000 is about 10^−5^ M.^42^ The concentration of C-PM (10^−2^ M) and PM (10^−2^ M) were much higher than CMC. Therefore, C-DVM and DVM in the sample are supposedly in the form of micelle. In phosphate-buffered saline (PBS), C-DVM was dispersed uniformly, and no significant change in the diameter was observed over six days, indicating the high stability of these micelles (Fig. 1c). The results of TEM imaging and the stability characterization of nonfunctionalized DVM are shown in Fig. S2. The doxorubicin drug release behavior of C-DVM in PBS in different pH conditions (pH 5.6 and pH 7.4) was separately examined by high-performance liquid chromatography (HPLC) (Fig. 1d). C-DVM released only 49.2% of D over a period of 24 h in PBS at pH 7.4, indicating that C-DVM could maintain the micelle structure stability under normal physiological conditions. In comparison, D was released more rapidly in a weakly acidic environment (pH 5.6) simulating the tumor microenvironment in vivo, with nearly 100% of D released within 24 h. All these data demonstrated the efficient assembly of C-DVM and its stability, uniformity and potential for targeted drug delivery to tumors.

**Table 1:** Size, PDI, and Zeta potential of DVM, C-DVM and C-PM

	Micelle size (nm)	PDI	Zeta potential (mV)
DVM	11.5 ± 0.40	0.258 ± 0.009	−7.30 ± 0.45
C-DVM	11.7 ± 0.53	0.268 ± 0.021	−7.68 ± 0.53
C-PM	9.4 ± 0.30	0.217 ± 0.014	−19.37 ± 0.42

### Effects of C-DVM on cellular uptake and microtubule dynamics inhibition

The murine mammary carcinoma cell line 4T1 was used for cellular drug uptake tests in vitro. We first sought to evaluate whether the drugs, D and V, which were encapsulated in C-DVM, could be efficiently delivered into cells. We compared a solution of free D and V (D + V group), a free D solution (DOX group), and DVM micelles (DVM group).

**Fig. 2:**
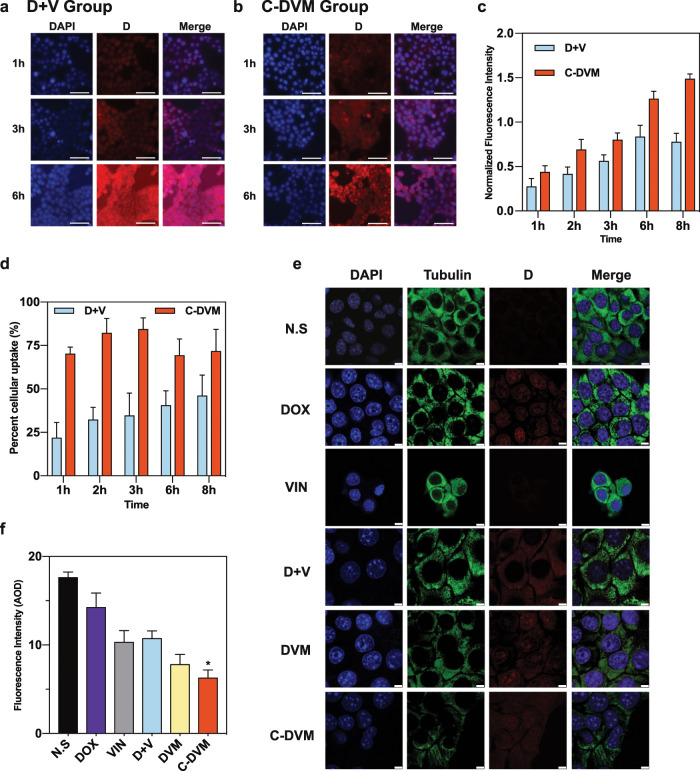
Drug internalization and microtubule disruption by C-DVM. **a** Representative micrographs of cellular uptake of D in D + V group and (**b**) C-DVM group at 1, 3, and 6 h incubation. Red symbolized the doxorubicin. DAPI stains nucleus (blue), scale bar represents 20 μm. **c**–**d** Quantification of cellular uptake and percentage of D in D + V and C-DVM groups. **e** Effects on the microtubule network in C-DVM and other groups, scale bar represents 7.5 μm. **f** Quantification of tubulin fluorescence in C-DVM and other groups. Nucleus and tubulin were stained with DAPI (blue) and Alexa Fluor 488-labeled anti-tubulin antibody (green), respectively. Data were presented as mean ± SD (*n* = 3), ^*^P *<* 0.05 vs D + V group

As shown in Fig. 2a, b, fluorescence microscopy showed significant doxorubicin uptake in cells treated with C-DVM at 3 h, compared with low uptake in the D + V group. These phenomena showed the desynchronized uptake of free drugs and drug-loaded micelles, which was also observed after 6 h of incubation. Comparable results were observed in DOX-treated cells (Fig. S3A) and DVM-treated cells (Fig. S3B). Next, we quantified the percentage of encapsulated drug that had been internalized (Fig. 2c, Fig. S2C). The results showed that C-DVM treatment led to increased drug delivery over time. In contrast, free D and V were found in the cytoplasm initially and then gradually disappeared after 6 h (Fig. 2c). Meanwhile, quantification of the normalized fluorescence intensity showed the low efficiency of cellular delivery in the D + V group (Fig. 2d). Similar differences in the delivery efficiency were also observed between the DOX group (Fig. S3C) and the DVM group (Fig. S3D). Therefore, we concluded that C-DVM could result in efficient cellular delivery and drug uptake.

Next, we investigated the ability of C-DVM to disrupt microtubules through the delivery of V. Figure 2e shows that the microtubule network exhibited normal organization in untreated 4T1 cells (N. S group) and in cells treated with free doxorubicin (DOX group) for 24 h, as visualized with immunofluorescence. Cells in the free vinorelbine (V) group and the D + V group showed moderate microtubule disruption. Quantification of the average optical density (AOD) of microtubule fluorescence in these two groups was performed (Fig. 2f), which showed a significant decrease in the AOD in both groups compared to that in untreated cells. In comparison, cells treated with C-DVM had low intensity in the microtubule network (Fig. 2e). We concluded that the vinorelbine loaded in the C-DVM with high efficacy cellular uptake led to tubulin expression decline and converted the cytoarchitecture due to insufficient microtubule structure. Microtubule fluorescence quantification showed that the micelle delivery of vinorelbine led to increased efficacy in disrupting microtubule assembly relative to that in the D + V group (Fig. 2f).

### Chemotherapeutic cytotoxicity and anti-migration & invasiveness abilities of C-DVM in vitro

**Fig. 3:**
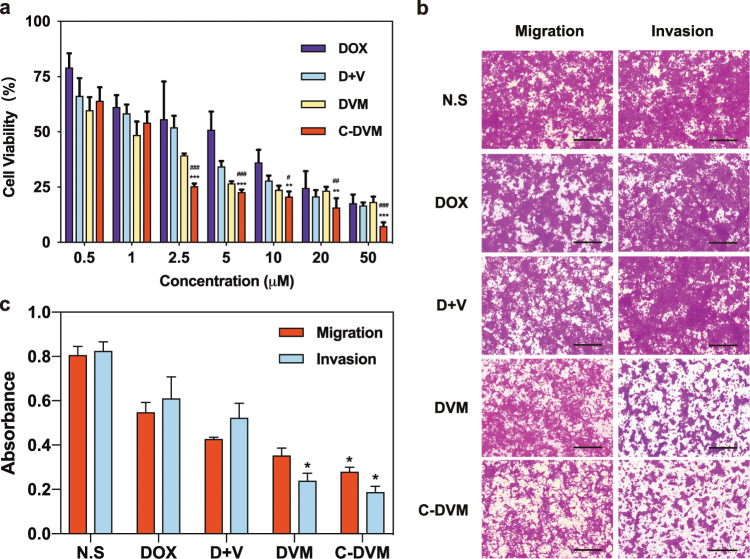
In vitro cytotoxicity and inhibitory effects of C-DVM on cell migration and invasion abilities in 4T1 cells. **a** The effect of different concentration of C-DVM and other treatments on cell viability of 4T1 cell line at 48 h measured by CCK-8. Error bars represent the mean ± SD (*n* = 5) of three independent experiments. ^*^P < 0.05, ^**^P < 0.01,^***^P < 0.005 vs D + V group, ^#^*P* < 0.05, ^##^*P* < 0.01,^###^*P* < 0.005 vs DVM group. **b** Representative images of migration and invasion assessments of DOX (free doxorubicin), D + V (the same dose of free doxorubicin and vinorelbine), DVM, and C-DVM (2.5 μM) in 4T1 cells. Scale bar represents 100 μm. **c** Quantification of the absorbance of migration and invasion activity for 4T1 cell line. Data were presented as mean ± SD (*n* = 5), ^*^P < 0.05 vs D + V group

The synergistic cytotoxicity of C-DVM combined with other solutions toward 4T1 cancer cells was evaluated by cell counting kit-8 (CCK-8) assays. Treatment with D + V resulted in 4T1 cell viability that was 58.4% and 52.1% of that of the untreated control at concentrations of 1 μM and 2.5 μM, respectively (Fig. 3a). In comparison, treatment with C-DVM led to a significant reduction in cell viability from 54.1% to 25.4% at the same concentrations. Furthermore, we found that little decline in viability was caused by C-DVM treatment at concentrations ranging from 2.5 μM to 20 μM, while it was reduced to 7.41% at 50 μM. We therefore used a treatment concentration of C-DVM of 2.5 μM in subsequent experiments. To rule out the effect of the potential cytotoxicity of C-PM on cell viability, we prepared a series of corresponding concentration solutions according to the D concentration, and the results showed little cytotoxicity (Fig. S4) in 4T1 cells.

Then, we evaluated the effect of C-DVM on migration and invasiveness. 4T1 cell migration and invasion were inhibited by C-DVM treatment, and the quantification showed a 22.7% reduction in invasiveness compared to that of untreated cells and a 41.6% reduction in invasiveness compared to that of the D + V group cells (Fig. 3b, c). The significant difference between the C-DVM and D + V groups illustrated the increased treatment efficacy of micelle-based codelivery for C-DVM treatment in inhibiting 4T1 cell invasion in comparison with that of the free drug. Migration was also poorly inhibited by the free drug treatments, such as D + V, but was significantly inhibited in C-DVM-treated cells (Fig. 3b, c), suggesting that C-DVM may be effective in inhibiting tumor cell metastasis. Taken together, the results showed that the incomplete eradication of highly invasive cancer cells such as 4T1 cells, which is a limitation of conventional chemotherapies such as D, could be overcome by the powerful potential of C-DVM. Therefore, we sought to explore C-DVM as a prospective agent for forward therapeutics.

### Pharmacokinetics study and biodistribution of C-DVM

**Fig. 4:**
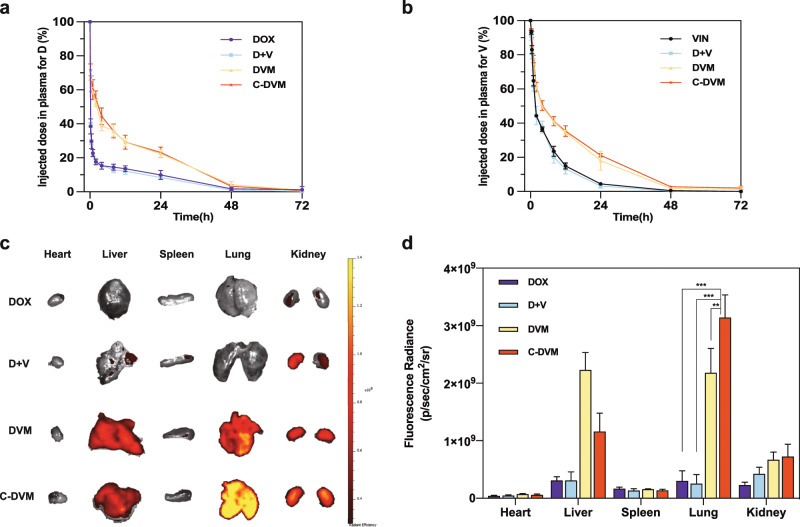
The study of pharmacokinetics and biodistribution of C-DVM. The pharmacokinetics profiles of (**a**) D and (**b**) V after intravenous delivery. All data were presented as mean ± SD (*n* = 5). **c** Doxorubicin accumulation in major organs in DOX, D + V, DVM, and C-DVM treatment groups, measured by doxorubicin fluorescence. **d** Quantification of the fluorescence radiance from c. All data were presented as mean ± SD (*n* = 5), ^**^P < 0.01, ^***^P < 0.005

The blood pharmacokinetics of C-DVM was studied in healthy BALB/C mice with other treatments as controls at designed timepoints. And the blood plasma concentration of doxorubicin and vinorelbine was measured by fluorescence and HPLC. The percentage of the injected dose of two drugs (Fig. 4a, b) illustrated that compared with the pharmacokinetics of D and V in D + V treated mice (*t*_1/2_ (D) = 0.64 h, *t*_1/2_ (V) = 1.82 h), D and V in C-DVM-treated mice stayed in circulation for a longer time (*t*_1/2_ (D) = 2.35 h and *t*_1/2_ (V) = 3.82 h). At 2 h after injection, approximately 51.48% of D and 58.69% of V remained in the blood circulation in the C-DVM group, whereas approximately 18.36% of D and 45.30% of V were reserved in the blood circulation with D + V group treatment at 2 h. Therefore, we concluded that C-DVM could prolong the period of drug availability and maintain high stability in blood, suggesting that C-DVM delivered by intravenous infusion could lead to drug delivery at metastatic tumor sites, for achieving synergistic targeted drug activity in vivo.

Next, to evaluate drug delivery by C-DVM in vivo, we generated a 4T1 breast cancer lung metastasis tumor model by injecting luciferase-expressing 4T1-luc cells. Observation of fluorescent foci in the lungs of the model mice by in vivo fluorescence imaging indicated that the metastatic model was generated successfully. Twenty-four hours after intravenous drug administration, the major organs of the sacrificed mice were collected for ex vivo imaging (Fig. 4c). As a measure of delivery efficiency, the doxorubicin fluorescence distribution in the different organs was quantified (Fig. 4d). The micelle groups (C-DVM and DVM) had much higher fluorescence intensity in lung metastatic sites than the free drug solution groups (DOX and D + V), implying that small micelles may show an advantage in drug delivery to metastases through the EPR effect. Moreover, compared to DVM, C-DVM had significantly greater lung metastatic site enrichment. We hypothesized that the CREKA peptides played an active role in targeting micelles to the tumor site. Meanwhile, the fluorescence signal was detected in the liver in the DVM and C-DVM group, suggesting part of the micelle accumulated in the liver. According to the fluorescence radiance, less than 25 percent of C-DVM was off target, while more than half of DVM was ineffectiveness. It reflected that C-DVM had better delivery efficacy than DVM. We also noticed increased kidney fluorescence in micelle groups, suggesting that micelles are metabolized out of the body through the kidney.

### Metastatic tumor inhibition due to targeted delivery in vivo

**Fig. 5:**
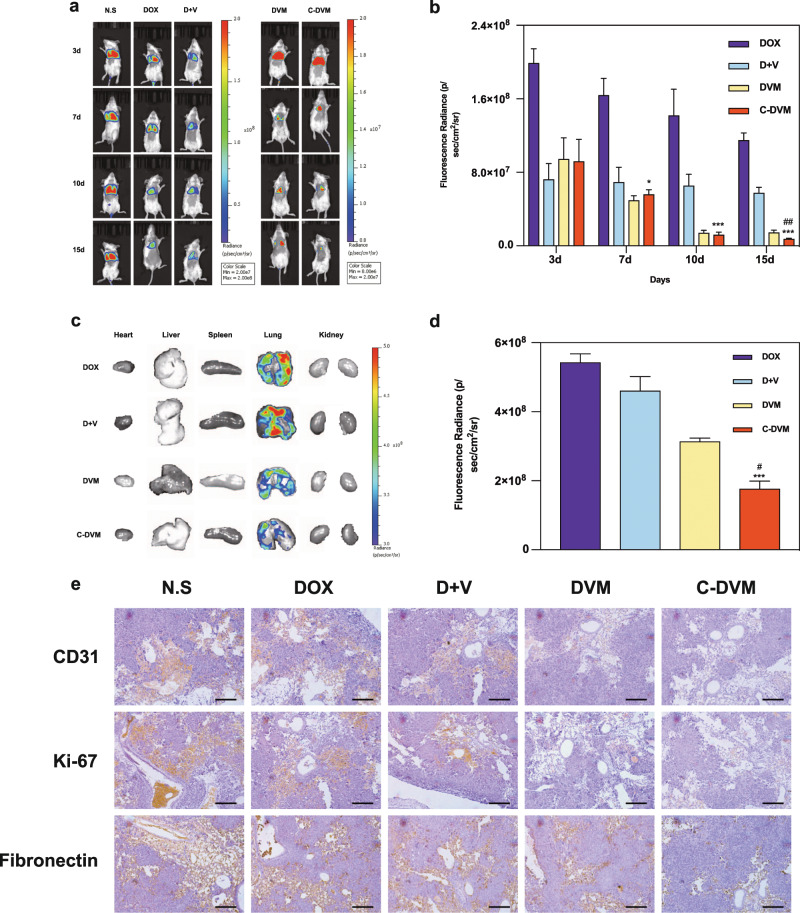
Tumor invasion inhibition in lung metastatic model of breast cancer. **a** Representative images of in vivo fluorescence of lung metastatic foci at different timepoints during treatment process, and (**b**) the quantification of fluorescence radiance based on in vivo images. **c** Representative images of ex vivo luciferase fluorescence imaging of major organs in all groups, and (**d**) the quantification of fluorescence radiance in lung metastatic foci based on ex vivo images. **e** Representative micrographs of H&E and IHC staining for CD31, Ki-67, and Fibronectin in tumor sections after treatments. Scale bar represents 20 μm. All data were presented as mean ± SD (*n* = 5), ^*^P < 0.05 vs D + V group, ^***^P < 0.005 vs D + V group. ^*#*^*P* < 0.05 vs DVM group, ^*##*^*P* < 0.01 vs DVM group

We next generated a lung metastasis model and visualized tumor growth by imaging fluorescence (IF) to evaluate the inhibition of metastatic tumor growth by C-DVM. Drug treatments were delivered by intravenous injection every two days for fifteen days. The mouse body weight and survival curves for each experimental group were recorded. By using luciferase fluorescence imaging to visualize metastatic tumor growth, we found that C-DVM treatment led to significant reduction in fluorescence after fifteen days of treatment (Fig. 5a), suggesting that effective drug delivery by C-DVM to the metastatic site could reduce the tumor size. Quantification of the fluorescence intensity in metastatic sites showed a reduction of 90% in C-DVM-treated mice (Fig. 5b) compared to that in the free D + V group. Moreover, while the C-DVM and DVM groups showed similar therapeutic effects during the first 7 days of treatment, after 15 days of treatment, the fluorescence intensity in the C-DVM group was half of that in the DVM group, which suggested that C-DVM treatment showed significantly higher efficacy in suppressing metastatic tumors compared to DVM at the end of treatment. Figure 5c shows the ex vivo imaging of the main organs excised from sacrificed mice after fifteen days of treatment. Correspondingly, C-DVM delivered D and V efficiently and showed superior accumulation in metastatic foci. The luciferase fluorescence again confirmed that metastatic tumors had been established in the lungs. Meanwhile, the reduction in invasive metastatic foci was apparent in micelle-treated (C-DVM and DVM) mice. Based on fluorescence quantification of metastatic foci (Fig. 5d), metastatic tumor growth in the C-DVM group was approximately one-third of that in the D + V group, which showed that C-DVM had exceptional performance in inhibiting the growth of lung metastatic foci. Comparing the lung metastasis fluorescence intensity between the C-DVM and DVM treatment groups showed that the C-DVM group had significantly lower intensity compared to the DVM group, reflecting a 2-fold reduction in tumor metastasis by C-DVM compared to that by DVM, which is consistent with the in vivo fluorescence quantification shown in Fig. 5b. Meanwhile, the survival (*n* = 8) (Fig. S5) and body weight variation curves (Fig. S6) showed that C-DVM resulted in improved health outcomes in a lung metastatic breast cancer mouse model. C-DVM outperformed the free drug in reducing metastatic invasion, as shown by the significantly decreased lung weight (Fig. S7).

To investigate how C-DVM achieved the improved inhibition of metastasis, we performed immunohistochemical (IHC) and hematoxylin-eosin (H&E) staining of CD31, Ki-67, and fibronectin in lung metastases (Fig. 5e). CD31 expression reflected the number of endothelial cells, which indicated that tumors promoted angiogenesis. According to IHC imaging, CD31 expression was reduced in the treatment groups. In particular, C-DVM had the lowest expression of CD31, implying a lack of angiogenesis, which may inhibit the growth of metastatic foci. Additionally, Ki-67 staining, a biomarker of tumor proliferative potential, showed relatively large numbers of Ki-67-positive cells in the D + V group and even more in the DOX group. Conversely, there were relatively few Ki-67-positive cells in the groups treated with C-DVM and DVM. Therefore, we could infer from tumor marker staining that the proliferation of metastatic foci was restricted by drug-loaded micelles. Meanwhile, we observed that the CREKA-functionalized micelle resulted in low degree fibronectin expression alongside low-level CD31 and Ki-67 expression, while high-level expression of fibronectin was observed in DOX and D + V treated groups, implying the metastatic sites contained highly invasive tumor cells rather than the low invasive potential of metastatic site tumor cells in the C-DVM group. Moreover, we focused on fibronectin expression between C-DVM and DVM. There was approximately equal expression of fibronectin between the DVM and D + V groups, which suggested that the drug-loaded micelles lacking CREKA peptides had low efficiency in inhibiting fibronectin expression, while the D + V groups showed high efficiency, which meant that CREKA peptides contributed to the inhibition of fibronectin expression by C-DVM.

### Safety evaluation of C-DVM treatment

**Fig. 6:**
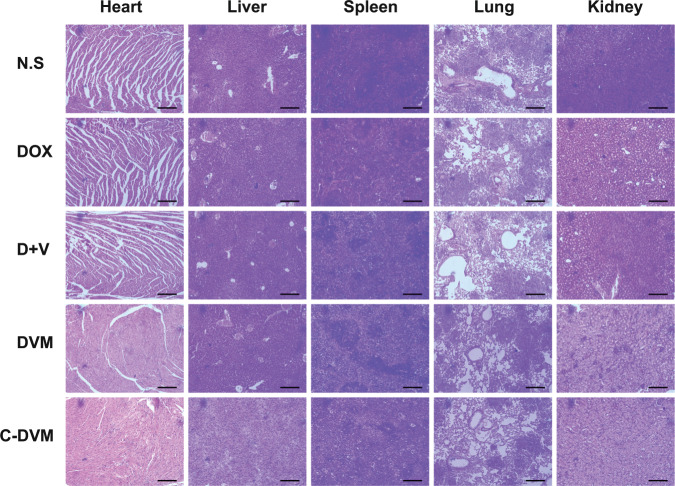
Representative micrographs of H&E staining in major organs of mice after various treatments. Scale bar represents 20 μm

To investigate whether C-DVM had potential side effects during treatment, the toxicity of C-DVM was evaluated in healthy mice. H&E staining was used to examine the effects of C-DVM administration on major organ histopathology (Fig. 6). Micrographs showed neither noticeable damage nor inflammation, suggesting that C-DVM had relatively low toxicity and few pathological effects on normal tissues. On the other hand, while mice showed significant body weight loss during the process of generating the tumor metastatic model (Fig. S6), the body weight stabilized during C-DVM interventional therapy, suggesting that mice with 4T1 tumor metastasis tolerated C-DVM treatment. Therefore, C-DVM could be regarded as a potential and efficacious therapeutic method for the inhibition of metastatic tumor invasion in vivo.

## Discussion

In summary, we have successfully developed a promising anti-metastatic therapy for inhibiting breast cancer metastatic tumor invasion by assembling targeted micelles functionalized with CREKA peptide that could codeliver doxorubicin and vinorelbine (C-DVM). C-DVM possessed high drug-loading capacity and a controlled small size and showed highly stable codelivery of two chemotherapy drugs, and it could efficiently deliver drugs to metastatic foci. Moreover, the good stability and tolerability of C-DVM could be used as a blueprint for the development of other agents for clinical use. Compared to the current gold standard chemotherapy (D + V), C-DVM could significantly inhibit metastatic foci growth and invasion through its highly efficient codelivery of both drugs to the tumor site. Last but not least, CREKA peptide functionalization showed an ability to enrich C-DVM in metastatic foci (Fig. 4c), which led to improved health outcomes compared to those in mice treated with nonpeptide-functionalized micelles (DVM). One interpretation based on past research^45^ is that the CREKA peptide contributed to micelle enrichment in fibronectin-expressing, highly invasive metastatic sites by achieving active targeting to tumors.

Ex vivo immunohistochemical staining demonstrated the anti-invasive effects of C-DVM on metastatic foci. Our results (Fig. 5e) showed that C-DVM treatment led to suppression of CD31 (a marker of angiogenesis), Ki-67 (related to tumor proliferation potential) and fibronectin (related to metastatic foci invasion), which led to a reduction in metastatic foci. Therefore, a therapeutic treatment based on dual-acting C-DVM could be highly effective for inhibiting metastatic invasion activity in breast cancer.

However, this study is limited by the fact that the metastatic mouse model may not completely recapitulate breast cancer metastasis in patients. Therefore, for stage IV breast cancer patients, it is predicted that C-DVM, as a candidate drug, should be further evaluated to develop therapeutics and consequently improve outcomes.

## Materials and methods

### Materials

Doxorubicin (doxorubicin hydrochloride, D) (Dalian Meilun Biotechnology Co., Ltd) and vinorelbine (vinorelbine tartrate, V) (Aladdin, Tianjin, China) were weighted by the analytical balance, then suspended in double-distilled water at 5 mM. DSPE-PEG-2000 (A.V.T (shanghai) Pharmaceutical Co., Ltd.), and DSPE-PEG-MAL (Mw 3200, MeloPEG Co., Ltd. Shanghai, China) were stored at −20 °C. CREKA peptide (Taopu Biotechnology, Shanghai, China) was stored at −20 °C.

### Synthesis of DSPE-PEG-2000-CREKA

CREKA (Mw 647) peptide (4.206 mg, 0.0065 mmol) and DSPE-PEG-MAL (16.0 mg, 0.0050 mmol) were dissolved in 5 mL 10% methanol purged with nitrogen. After stirring for 4 h at room temperature, the solution dialyzed (molecular weight cutoff of Mw 1000) against double-distilled water for 36 h. The resulting DSPE-PEG-2000-CREKA (DPC) was lyophilized for future use.

### Preparation of micelles

C-DVM was prepared by chemotherapy agents and DPC. D and V are water-soluble drugs, which are encapsulated into the micelle via a one-step self-assembly method.^51^ C-DVM was prepared with the equal molar ratio of D and V incorporated into DPC aqueous solution (1:1:2, m/m/m), and incubated at room temperature with ultrasound mixing at 120 W for 30 min. Centrifugation was used to remove free D and V by using a filter (molecular weight cutoff of Mw 3000). DVM assembly follows a similar procedure using DSPE-PEG-2000 in place of DPC. Then, the incubated solution was filtered using cellulose acetate membranes (0.22 µm pore size, Beijing Solarbio Science & Technology Co., Ltd, China). The released products and incorporated in micelles of D were detected by using ultraviolet spectrophotometry and RP-HPLC.

### Evaluation of the physicochemical characterization of C-DVM

The DLS analyzer (Brookhaven Instruments Co., USA) was used to measure the basic properties of C-DVM such as size, polydispersity index (PDI) and zeta potential. In order to more precisely examine the size of C-DVM micelles, the C-DVM solution was diluted then stained with 10% phosphotungstic acid to acquire TEM images (TECNAI F20, FEI, USA). The shape and surface topology of C-DVM were collected by AFM (JPK nanowizard II, Germany). Briefly, 0.5 μmol C-DVM solution was dispersed in deionized water in an ice bath with sonication. Taking a drop of diluted solution placed on the purged single polishing high purity silicon wafer for imaging.

The peak of D or V was measured by HPLC (1260 Infinity, Agilent, USA). Entrapment efficiency (EE) of D or V calculated by the equation:

1 $${\mathrm{EE}}\,\left( {\mathrm{\% }} \right) = \frac{{{\rm{Weight}}\;{\rm{of}}\;{\rm{drug}}\;{\rm{encapsulated}}\;{\rm{in}}\;{\rm{C-DVM}}}}{{{\rm{Initial}}\;{\rm{weight}}\;{\rm{of}}\;{\rm{drug}}}} \times 100\%.$$

Briefly, 0.22 µm cellulose acetate membrane was used to filter the samples followed by additional dilution in acetonitrile with an eluant solution via the C18 column to detect the leakage of D, as previously reported.^52^ The leakage of V in C-DVM was diluted by acetonitrile with an eluant solution via the C18 column, as previously reported.^53^

To evaluate the drug release kinetics, 1 mL C-DVM solution was added to 1 mL fresh serum of mouse. The solution dialyzed (molecular weight cutoff of Mw 1000) and incubated in 50 mL of either acetate buffer (pH 5.6) or phosphate buffer (pH 7.4) with gentle shaking at 37 °C to simulate drug release in vitro. HPLC was used to measure the amount of released D. All experiments were done in triplicate. The calculation of doxorubicin release from C-DVM was calculated by

2 $${\mathrm{cumulative}}\;{\mathrm{amount}}\;{\mathrm{released}}\,\left( {\mathrm{\% }} \right) = \frac{{{\rm{Concentration}}_{{\rm{drug}}\;{\rm{released}}}}}{{{\rm{Concentration}}_{{\rm{drug}}\;{\rm{contained}}}}} \times 100\%.$$

### Cell culture

The mouse mammary carcinoma cell line 4T1-luc was purchased (American Type Culture Collection, ATCC; Manassas, VA). D-luciferin potassium salt was used for the substrate of 4T1-luc cells to observe fluorescence. The DMEM medium (P0201-07, Biogene, China) including 10% fetal bovine serum (Invitrogen, Carlsbad, CA) was used to culture 4T1-luc cells.

### Cellular uptake by fluorescence imaging

During the treatment, 4T1 cells were observed at 1, 2, 3, 6, and 8 h, then fixed with 4% paraformaldehyde (PFA) for 15 min and washed one more time. PBS thrice was used to wash treated cells. Fluorescence microscopy (Leica DM2500, Germany) was used to detect doxorubicin (ex. = 485 nm, em. = 530 nm) fluorescence signal. 4′,6-diamidino-2-phenylindole (DAPI, ex. = 364 nm, em. = 454 nm) was used for staining nuclei to make cell localization. The fluorescence signal was measured with Image J software. The initial drug fluorescence signal was measured at the beginning. The percentage of uptake drug was calculated by

3 $${\mathrm{ratio}}\;{\mathrm{of}}\;{\mathrm{cellular}}\;{\mathrm{uptake}}\;{\mathrm{drug}}\,\left( {\mathrm{\% }} \right) = \frac{{{\rm{Fluorescence}}\;{\rm{signal}}_{{\rm{cellular}}\;{\rm{uptake}}\;{\rm{drug}}}}}{{{\rm{Fluorescence}}\;{\rm{signal}}_{{\rm{initial}}\;{\rm{drug}}}}} \times 100\%.$$

### Immunofluorescence study in vitro

4T1 cells were treated with 100 nM free vinorelbine (VIN group), DVM, or C-DVM for 24 h then washed with PBS, the solution was fixed in 4% PFA. 0.3% Triton-X 100 and blocked with 5% bovine serum albumin in PBS was added to the solution for permeabilizing cells for 1 h at room temperature, then incubated with 5% goat nonimmune serum. Primary anti-*α*-tubulin polyclonal antibody (1:200, 1 μg/mL, Beijing Solarbio Science & Technology Co., Ltd.) was incubated at 4 °C overnight. The goat anti-mouse immunoglobulin G (H + L) conjugated with Alexa Fluor 488 (1:200, 2 μg/mL, Invitrogen) protected from light at room temperature was used for the secondary detection for tubulin. Finally, the cells were stained with DAPI for 30 min to visualize nuclei. Between each step, cells were rinsed with PBS 3 times.

### Confocal microscopy

4T1 breast cancer cells were seeded onto 35 mm high-affinity culture dishes (D35-20-1.5P, Cellvis, Mountain View, CA) and incubated for 24 h (1 × 10^5^ cells /well). Fresh cell culture media containing C-DVM or other groups was added for another 24 h. The nuclei were stained with DAPI after three washes and observed with Laser Scanning Confocal Microscope 710 confocal microscope (Carl Zeiss, USA) at ×100 magnification.

### Cell cytotoxicity

Cell counting kit-8 (CCK-8) assay (70-CCK801, MultiSciences, CA) was used to evaluate the cytotoxicity of C-DVM against 4T1 cells. 4T1 cells treated with DOX (free doxorubicin), D + V (the same dose of doxorubicin and vinorelbine), DVM, and C-DVM at the equivalent concentration as experiment groups in DMEM medium for 48 h and the same volume of PBS as the control group. Each group was set in triplicate. After that, the media was dealt with CCK-8 protocol. The cell viability ratio, for each treatment group, was calculated as a percentage of the control group.

### Migration and invasion

For cell migration assay, 200 μL cell suspension was seeded in the upper chambers, while 800 μL completed medium was seeding in the lower chambers in Trans-well plate with Transparent PET Membrance (24-well, 8.0 μm pore size, Corning, USA). When the cells attached, the medium with C-DVM or other solutions was treated.

For cell invasion assay, 100 μL cell suspension with 2.5 μM C-DVM in medium without serum was added into the upper chamber, medium with 10% FBS as chemoattractant was added to the lower chamber in the Matrigel Invasion Chamber (24-well, 8.0 μm pore size, Corning, USA). Cells were incubated for 24 h, then 4% PFA was used to fix with the upper chambers for 15 min. The migrated cells located in the lower chamber stained with crystal violet then counted in the field of the microscope.

### Plasma pharmacokinetics

Whole blood in four groups (n = 5/group) were drawn from female Balb/c mice (6 weeks old, Beijing Vital River Laboratory Animal Technology Co., Ltd) after intravenous injection. DOX (5 mg doxorubicin/kg), D + V (5 mg doxorubicin/kg, 7.88 mg vinorelbine/kg), DVM (5 mg doxorubicin/kg, 7.88 mg vinorelbine/kg) and C-DVM (5 mg doxorubicin/kg, 7.88 mg vinorelbine/kg). After administration, approximately 300 μL of blood was collected from each mouse at the estimated timepoints from the orbital venous plexus using heparinized tubes by retro-orbital venipuncture. Plasma samples were harvested by immediate centrifugation (4000 rpm, 12 min) and stored at −20 °C, then preprocessed by acetonitrile and precipitated by centrifugation (−20 °C, 12 000 rpm, 12 min). Next, 100 μL of supernatant was removed into black 96-well plates and the fluorescence intensity of doxorubicin was detected by a microplate reader. Meanwhile, the concentration of vinorelbine was measured by HPLC-VWD.

### In vivo biodistribution study and fluorescence imaging of C-DVM

The established lung metastasis model mice were intravenous injected with C-DVM (5 mg doxorubicin/kg, 7.88 mg vinorelbine/kg) and other experiment groups (*n* = 5/group). After the mouse sacrificed, main organs excised from each sacrificed mouse for fluorescence imaging after 24 h administration, then analyzed on the IVIS Imaging Spectrum System (IVIS, PerkinElmer) under parameters for doxorubicin fluorescence mentioned above.

### Establishment of lung metastasis model of breast cancer and metastatic tumor growth inhibition activity in vivo

Five groups (*n* = 8/group) of female Balb/c mice were injected with 4T1-luc cells (1 × 10^6^ cells/mouse) via tail vein to establish the metastasis model. The mice body weight data were recorded every other day. Three days after the metastasis model established, the mice were injected with C-DVM (35 mg/kg body weight) or other drug solutions every two days by tail intravenous injection, while the N.S group was injected with saline. At certain timepoints (3 d, 7 d, 10 d, 15 d), all groups were injected with D-luciferase (150 mg/kg) (Aladdin, USA). At 15 min after injection, the fluorescence signal was monitored by IVIS. In order to further evaluate the metastatic foci, the major organs excised from sacrificed mice were imaged.

### Histological analysis

The mice were sacrificed and dissected after seven treatments. Hematoxylin and eosin (H&E) staining was used to stain major organs excised from mice to evaluate the toxicity of various drug formulations. Additionally, immunohistochemistry (IHC) staining was used to label the lung metastasis tissue slices in different treatment groups to analyze the metastatic foci angiogenesis, proliferation, and invasion biomarker expression. IHC and H&E micrographs were imaged (Leica DMI3000B, Germany). The Anti-mouse CD31 antibody, Anti-rabbit Ki-67 antibody, and Anti-rabbit Fibronectin antibody were used for immunostaining.

### Safety evaluation

Five groups of healthy female Balb/c mice (*n* = 5/group) were intravenously injected with free doxorubicin (DOX), free doxorubicin and vinorelbine (D + V), DVM, or C-DVM (5 mg doxorubicin/kg) as experiment group and 0.9% saline as N.S group every other two days. In the end, the sacrificed mice major were excised for histologic study by H&E staining.

### Statistical analysis

Statistical analysis was carried out by student’s t-test to test the differentiation between two groups, while one-way analysis of variance (ANOVA) was taken for variance in multiple (>3) groups. Significant difference was considered when *p < 0.05, **p < 0.01, and ***p < 0.001. All data were shown as mean ± standard deviation (SD).

## Supplementary information

Supplementary Information-Fibronectin-Targeted Dual-acting Micelles for Combination Therapy of Metastatic Breast Cancer

## References

[1] Cronin KA (2018). Annual report to the nation on the status of cancer, part I: national cancer statistics. Cancer.

[2] Weigelt B, Peterse JL, van ‘t Veer LJ (2005). Breast cancer metastasis: markers and models. Nat. Rev. Cancer.

[3] Hameed S (2019). Fluorescence guided sentinel lymph node mapping: from current molecular probes to future multimodal nanoprobes. Bioconjugate Chem..

[4] Cao H (2015). Hydrophobic interaction mediating self-assembled nanoparticles of succinobucol suppress lung metastasis of breast cancer by inhibition of VCAM-1 expression. J. Control Release.

[5] Persidis A (1999). Cancer multidrug resistance. Nat. Biotechnol..

[6] Gao C (2018). Amphiphilic drug conjugates as nanomedicines for combined cancer therapy. Bioconjugate Chem..

[7] Szakacs G, Paterson JK, Ludwig JA, Booth-Genthe C, Gottesman MM (2006). Targeting multidrug resistance in cancer. Nat. Rev. Drug Discov..

[8] Aguirre-Ghiso JA (2007). Models, mechanisms and clinical evidence for cancer dormancy. Nat. Rev. Cancer.

[9] Furedia A (2017). Pegylated liposomal formulation of doxorubicin overcomes drug resistance in a genetically engineered mouse model of breast cancer. J. Control Release.

[10] Jordan MA, Wilson L (2004). Microtubules as a target for anticancer drugs. Nat. Rev. Cancer.

[11] Dumontet C, Jordan MA (2010). Microtubule-binding agents: a dynamic field of cancer therapeutics. Nat. Rev. Drug Discov..

[12] Na GC, Timasheff SN (1980). Thermodynamic linkage between tubulin self-association and the binding of vinblastine. Biochemchemistry.

[13] Lobert S, Correia JJ (2000). Energetics of vinca alkaloid interactions with tubulin. Method Enzymol..

[14] Duflos A, Kruczynski A, Barret JM (2002). Novel aspects of natural and modified vinca alkaloids. Curr. Med. Chem. Anticancer Agents.

[15] Jassem J (2003). Oral vinorelbine in combination with cisplatin: a novel active regimen in advanced non-small-cell lung cancer. Ann. Oncol..

[16] Rossi A (2003). Single agent vinorelbine as first-line chemotherapy in elderly patients with advanced breast cancer. Anticancer Res..

[17] Seidman AD (2003). Monotherapy options in the management of metastatic breast cancer. Semin. Oncol..

[18] Plosker GL, Figgitt DP (2003). Rituximab—a review of its use in non-Hodgkin’s lymphoma and chronic lymphocytic leukaemia. Drugs.

[19] Sandler AB (2003). Chemotherapy for small cell lung cancer. Semin. Oncol..

[20] Armitage JO (2002). Overview of rational and individualized therapeutic strategies for non-Hodgkin's lymphomas. Clin. Lymphoma.

[21] Mickolajczyk KJ, Geyer EA, Kim T, Rice LM, Hancock WO (2019). Direct observation of individual tubulin dimers binding to growing microtubules. Proc. Natl Acad. Sci. USA.

[22] Martino E (2018). Vinca alkaloids and analogues as anti-cancer agents: looking back, peering ahead. Bioorg. Med. Chem. Lett..

[23] Aapro M (2019). Randomized phase II study evaluating weekly oral vinorelbine versus weekly paclitaxel in estrogen receptor-positive, HER2-negative patients with advanced breast cancer (norBreast-231 trial). Breast.

[24] Katselashvili L (2017). Paclitaxel every-3-weeks versus weekly paclitaxel and versus weekly vinorelbine in metastatic breast cancer. Ann. Oncol..

[25] Dieras V (1997). Docetaxel in combination with doxorubicin or vinorelbine. Eur. J. Cancer.

[26] Hameed S (2018). Self-assembly of porphyrin-grafted lipid into nanoparticles encapsulating doxorubicin for synergistic chemo-photodynamic therapy and fluorescence imaging. Theranostics.

[27] Spielmann M (1994). Phase-II Trial of vinorelbine doxorubicin as first-line therapy of advanced breast-cancer. J. Clin. Oncol..

[28] Nazir T, Islam A, Omer MO, Mustafa M (2015). Lymphocytopenia; induced by vinorelbine, doxorubicin and cisplatin in human cancer patients. Breast Dis..

[29] Wu CY (2017). Monitoring tumor response after liposomal doxorubicin in combination with liposomal vinorelbine treatment using 3′-Deoxy-3′-[^18^F] fluorothymidine PET. Mol. Imaging Biol..

[30] Norris B (2000). Phase III comparative study of vinorelbine combined with doxorubicin versus doxorubicin alone in disseminated metastatic/recurrent breast cancer: national cancer institute of Canada clinical trials group study MA8. J. Clin. Oncol..

[31] Lankelma J (1999). Doxorubicin gradients in human breast cancer. Clin. Cancer Res..

[32] Quail DF, Joyce JA (2013). Microenvironmental regulation of tumor progression and metastasis. Nat. Med..

[33] Reticker-Flynn NE (2012). A combinatorial extracellular matrix platform identifies cell-extracellular matrix interactions that correlate with metastasis. Nat. Commun..

[34] Pupa SM, Menard S, Forti S, Tagliabue E (2002). New insights into the role of extranucleus matrix during tumor onset and progression. J. Cell Physiol..

[35] Hynes, R O. *Interactions of Fibronectins. In: Fibronectins.* Springer-Verlag, Inc. (1990).

[36] Zhou Z (2015). MRI detection of breast cancer micrometastases with a fibronectin-targeting contrast agent. Nat. Commun..

[37] Malik G (2010). Plasma fibronectin promotes lung metastasis by contributions to fibrin clots and tumor cell invasion. Cancer Res..

[38] Kaplan RN, Rafii S, Lyden D (2006). Preparing the “soil”: the premetastatic niche. Cancer Res..

[39] Torchilin VP (2005). Recent advances with liposomes as pharmaceutical carriers. Nat. Rev. Drug Discov..

[40] You YJ (2018). Porphyrin-grafted lipid microbubbles for the enhanced efficacy of photodynamic therapy in prostate cancer through ultrasound-controlled in situ accumulation. Theranostics.

[41] Zhang NS (2018). Localized delivery of curcumin into brain with polysorbate 80-modified cerasomes by ultrasound-targeted microbubble destruction for improved Parkinson's disease therapy. Theranostics.

[42] Lukyanov AN, Gao ZG, Mazzola L, Torchilin VP (2002). Polyethylene glycol-diacyllipid micelles demonstrate increased accumulation in subcutaneous tumors in mice. Pharm. Res..

[43] Matsumura Y, Maeda H (1986). A new concept for macromolecular therapeutics in cancer- chemotherapy- mechanism of tumoritropic accumulation of proteins and the antitumor agent smancs. Cancer Res..

[44] Maeda H, Wu J, Sawa T, Matsumura Y, Hori K (2000). Tumor vascular permeability and the EPR effect in macromolecular therapeutics: a review. J. Control Release.

[45] Simberg D (2007). Biomimetic amplification of nanoparticle homing to tumors. Proc. Natl Acad. Sci. USA.

[46] Stein PD (2006). Incidence of venous thromboembolism in patients hospitalized with cancer. Am. J. Med..

[47] Bardos H, Molnar P, Csecsei G, Adany R (1996). Fibrin deposition in primary and metastatic human brain tumours. Blood Coagul. Fibrin.

[48] Chung EJ (2014). Fibrin-binding, peptide amphiphile micelles for targeting glioblastoma. Biomaterials.

[49] Zhou ZX, Wu XM, Kresak A, Griswold M, Lu ZR (2013). Peptide targeted tripod macrocyclic Gd(III) chelates for cancer molecular MRI. Biomaterials.

[50] Agemy L (2010). Nanoparticle-induced vascular blockade in human prostate cancer. Blood.

[51] Wang Y (2010). Pegylated phospholipids-based self-assembly with water-soluble drugs. Pharm. Res..

[52] Tang N (2007). Improving penetration in tumors with nanoassemblies of phospholipids and doxorubicin. J. Natl Cancer Inst..

[53] Liu ZB (2015). Aptamer density dependent cellular uptake of lipid-capped polymer nanoparticles for polyvalent targeted delivery of vinorelbine to cancer cells. RSC Adv..

