# Supplementary material for: Fibronectin-targeted dual-acting micelles for combination therapy of metastatic breast cancer
Source: Signal Transduct Target Ther. 2020 Feb 7;5:12. doi: 10.1038/s41392-019-0104-3 (PMC7005157; doi:10.1038/s41392-019-0104-3)
Supplement: Supplementary file 1 — Supplementary Information-Fibronectin-Targeted Dual-acting Micelles for Combination Therapy of Metastatic Breast Cancer [file 41392_2019_104_MOESM1_ESM.docx]

Supplementary Information

Fibronectin-Targeted Dual-acting Micelles for Combination Therapy of Metastatic Breast Cancer

Zhuoran Gong^1^, Min Chen^1^, Qiushi Ren^1^ Xiuli Yue^2*^ and Zhifei Dai^1*^

^1^ Department of Biomedical Engineering, College of Engineering, Peking University, Beijing, 100871, China.

^2^ School of Environment, Harbin Institute of Technology, Harbin 150090, China

* Correspondence and requests for materials should be addressed to X.Y. (Email: [xiulidx@163.com](mailto:xiulidx@163.com)) and Z.D. (Email: [zhifei.dai@pku.edu.cn)](mailto:zhifei.dai@pku.edu.cn))

Homepage: <http://mimit-pku.org>


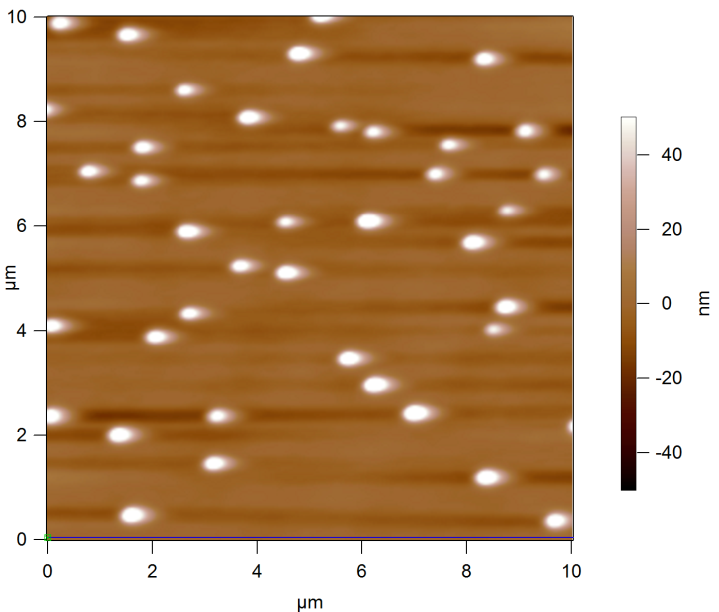


**Fig. S1.** Representative AFM imaging of C-DVM.

**Fig. S2.** **A)** TEM imaging of DVM. **B)** Stability test of DVM over six days in PBS.

**Fig. S3.** **A)** Representative micrographs of nucleus uptake of D in DOX group and **B)** DVM group in 1,2,3,6,8hours incubation. DAPI (blue) stains nucleus, scale bar represents 20μm. **C)** Quantification of the cell uptake of D mixed in DOX group and encapsulated in DVM, and **D)** quantification percentage of D nucleus uptake of DOX group and DVM group.

**Fig. S4.** In vitro cytotoxicity of a series of concentration of C-PM reflects on cell viability of 4T1 cell line at 48h. Error bars represent the mean ± SD (n=5) of three independent experiments.

**Fig. S5.** The survival curve of C-DVM and other groups during the treatment.

**Fig. S6.** Body weight variation of mice treated with different drug formulations. All data were presented as mean ± SD (n = 8).

**Fig. S7.** Lung weight of mice treated with different drug formulations. All data were presented as mean ± SD (n = 8).
